# P6 Electroacupuncture Improved QTc Interval Prolongation by Upregulation of Connexin43 in Droperidol Treated Rats

**DOI:** 10.1155/2014/926423

**Published:** 2014-10-13

**Authors:** Feng Zhao, Suyang Cui, Libing Huang

**Affiliations:** Department of Anesthesiology, Affiliated Hospital of Nanjing University of Chinese Medicine, Nanjing, Jiangsu 210029, China

## Abstract

*Aim*. This study investigated the effect of P6 EA on droperidol-induced QTc interval prolongation and Cx43 expression in ventricular muscle of rats. *Methods*. Twenty-four rats were randomly divided into control group (C), droperidol group (D), or EA group (E). C group rats were injected with normal saline. D group rats were injected with droperidol 0.13 mg/kg. E group rats were pretreated with EA at left P6 acupoint for 30 min and then injected with droperidol (0.13 mg/kg). QTc intervals were recorded at lead II in ECG within 120 min. Cx43 expression was measured by RT-PCR and western blotting. *Result*. Droperidol significantly prolonged QTc intervals compared with controls at 5 min, 10 min, 15 min, and 30 min (*P* < 0.05). P6 EA could significantly abbreviate the prolongation of QTc interval compared with droperidol group at 5 min, 10 min, 15 min, and 30 min (*P* < 0.05). Cx43 mRNA and proteins were significantly increased by P6 EA compared with droperidol group at 120 min (*P* < 0.05). There were no significant differences in Cx43 mRNA and protein expression between droperidol and control group at 120 min (*P* > 0.05). *Conclusion*. P6 EA could improve QTc interval prolongation induced by droperidol, which may relate to upregulation of Cx43 mRNA and protein. Antiemetic dose of droperidol had minor effects on Cx43 mRNA and protein expression at 120 min.

## 1. Introduction

Postoperative nausea and vomiting (PONV) is one of the most common complaints in the surgical patients, and its incidence still remains high [1]. Droperidol is one of the oldest and most controversial antiemetics used to treat PONV. In 2001, droperidol was issued with a “black box” warning regarding its potential QT interval prolongation and Torsades de Pointes by the US Food and Drug Administration (FDA), which was challenged by many anesthesiologists [2]. Therefore, nonpharmacological methods were considered in preventing PONV. However, P6 (Neiguan) acupuncture was effective in preventing and treating PONV with minor side effects [3]. Moreover, P6 acupuncture showed antiarrhythmic effects and this might benefit the QT prolongation induced by droperidol. Gap junctions protein connexin43 (Cx43) played important roles in QT interval prolongation and Torsades de Pointes [4].

Based on the clinical and experimental reports of its antiemetic and antiarrhythmic properties, this study was to determine the hypothesis that P6 EA may improve QT interval prolongation induced by droperidol via increasing Cx43 expression in Sprague-Dawley rats.

## 2. Materials and Methods

### 2.1. Animals and Model

The study was performed in accordance with the protocols approved by Nanjing University of Chinese Medicine on Animal Care and Use. Twenty-four adult female Sprague-Dawley rats weighing about 250 g were used and housed in the home cage at 24 ± 1°C under 12 h on/off light cycle. Free food and water were provided with humane care.

Rats were randomly divided into 3 groups: control (C), droperidol (D), and electroacupuncture (E) (eight rats per group). Before the experiments, rats were anesthetized by intraperitoneal injection of sodium pentobarbital (30 mg/kg). A 2 cm skin incision was made to expose the left femoral vein and one 24-gauge catheter was inserted into the femoral venous vessel under direct visualization for drug infusion. Then skin incisions were closed with suture. Rats were injected through femoral vein with droperidol (0.13 mg/kg) in D group and the equal volumes of saline in C group. Rats in E group were pretreated with electroacupuncture at left P6 acupoint for 30 min before droperidol injection.

### 2.2. Electroacupuncture

A 15 mm long and 0.3 mm diametrical acupuncture needle was inserted through the subcutis about 2 mm at left P6 acupoint. The acupoint was connected to the negative pole of EA apparatus (XS-998, Xiaosong, Nanjing), and the positive pole was connected to the left hand limb. The rats with EA were electrically stimulated with dense-sparse pulses, frequency of 2–10–100 Hz, and 1 mA strength for 30 min.

### 2.3. Electrocardiograph (ECG) Recording

ECG was invasively monitored and recorded with Dash4000 system (GE,USA) in Lead II at different time points: before injection and 5 min, 10 min, 15 min, 30 min, 60 min, and 120 min after injection. The QT intervals were measured and recorded as QTc adjusted with Bazett's formula QTc-B = QT/√(RR). Rats were sacrificed at 120 min after the ECG monitoring. The hearts were harvested and fast-frozen in liquid nitrogen for other tests.

### 2.4. RNA Extraction and Reverse Transcription-Polymerase Chain Reaction (RT-PCR) Analysis

To examine Cx43 mRNA expression, total RNA was extracted from fast-frozen left ventricular tissues with Trizol (Invitrogen, USA), and DNA-free RNA was prepared using DNase I (Fermentas, Lithuania) according to the instructions. Reverse transcription of RNA was performed with the RevertAid first-strand cDNA synthesis kit (Fermentas, Lithuania) according to the protocol. To amplify Cx43 from cDNA, the primers were designed by Primer 5 software (Source Forge) as 5-CAACTCCACGGGAACGAA-3 (forward primer) and 5-CAACTCCACGGGAACGAA-3 (reverse primer) and Rat-GAPDH primers as 5-TGTTGCCATCAACGACCCCTT-3 (forward primer) and 5-CTCCACGACATACTCAGCA-3 (reverse primer). PCR was carried out in mixtures containing 2 *μ*L of each primer (10 *μ*M), 5 *μ*L 10 × Taq buffer, 4 *μ*L MgCl_2_ (25 mM), 4 *μ*L dNTPs (10 mM), 1 *μ*L RevertAid reverse transcriptase, 0.5 *μ*L Taq polymerase (5 u/*μ*L) (Fermentas, Lithuania), and 2 *μ*L of extracted RNA and DEPC H_2_O up to a total volume of 50 *μ*L. PCR cycling scheme included 95°C for 5 min, 32 cycles of 95°C for 30 s, 57°C for 30 s, and 72°C for 45 s, followed by 72°C for 10 min. Products were separated on electrophoresis gel, stained with ethidium bromide, and analyzed with the Gel DOC XR Imaging System (Bio-Rad, USA).

### 2.5. Western Blotting Was Used for Detection of the Expression Cx43 Proteins in Hearts

Left ventricle tissues were homogenized in ice-cold lysis buffer (1% Triton X-100, 150 mM NaCl, 10 mM Tris, pH 7.4, 1 mM EDTA, 1 mM EGTA, pH 8.0, 0.2 mM sodium orthovanadate, 0.2 mM PMSF, and 0.5% NP-40). The resulting lysates were cleared by centrifugation at 13,000 ×g for 10 min. The lysate samples containing 70 *μ*g of total protein were electrophoresed on 10% SDS-polyacrylamide gels and transferred to nitrocellulose membranes. An immunoblot for GAPDH protein was used as a control for equal protein loading. Membranes were blocked at room temperature with BSA (3% wt/vol) in Tris-buffered saline containing Tween 20 (0.5% vol/vol; TBS-T) for 1 hr and then incubated with monoclonal antibody (1 : 1000 dilution; Immunoway, USA) to measure nonphosphorylated component of Cx43 overnight at 4°C. The membranes were washed and then incubated with horseradish peroxidase-conjugated goat anti-rat secondary antibody (1 : 1000 dilution). All antibodies were obtained from Lufei Laboratories (Nanjing, CHINA). Chemiluminescence reagent (ECL; Pierce Inc., Rockford, IL, USA) was used for immunodetection, and blots were exposed to autoradiographic X-ray film. Immunoreactivity was quantified by densitometric analysis with Image-Pro Plus 6.0 software (Media Cybernetics Inc., Silver Spring, MD, USA). Relative protein level was calculated as the ratio of the band density of Cx43 and that of GAPDH.

### 2.6. Statistical Analysis

All data were presented as mean ± SEM. Statistical analysis was performed with SPSS Statistics 17.0 (SPSS Inc., Chicago, IL, USA) software by using one-way analysis of variance (ANOVA) and *t*-test in different groups. *P* < 0.05 was considered statistically significant.

## 3. Results

### 3.1. Effect of P6 EA on Droperidol-Induced QTc Interval Prolongation

**Figure 1:**
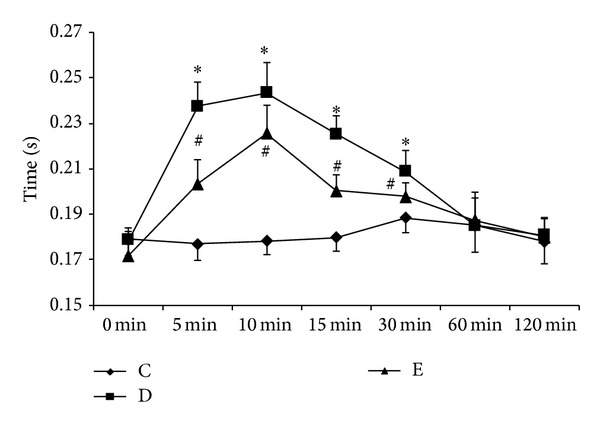
Effects of P6 EA on QTc prolongation induced by droperidol in rats. QTc intervals were analyzed in control (C), droperidol (D), and EA (E) groups at different time points. All values are expressed as mean ± SD; *n* = 8 for each group. ^#^ *P* < 0.05 versus droperidol group (D), *P < 0.05 versus control group (C).

As shown in Figure 1, QTc intervals were significantly increased after droperidol injection at 5 min, 10 min, 15 min, and 30 min (*P* < 0.05 versus C group), and P6 EA significantly abbreviated the QTc intervals prolongation at the above time points (*P* < 0.05 versus D group). Results indicate that EA exerts preventive properties against droperidol-induced QTc intervals prolongation.

### 3.2. Effect of P6 EA on the Expression of Cx43 mRNA in Hearts

**Figure 2:**
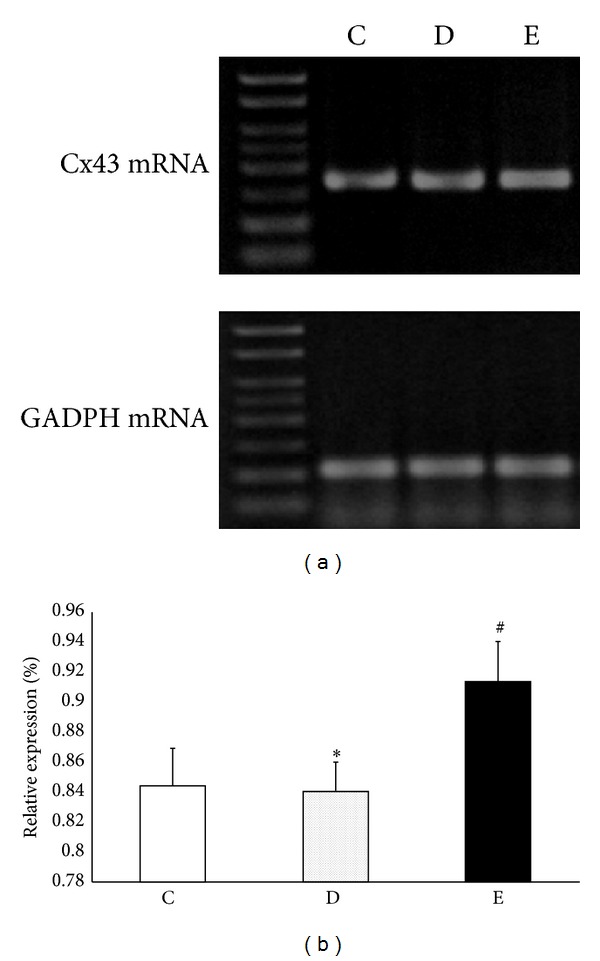
Effects of P6 EA on Cx43 mRNA expression in a rat model of droperidol-induced QTc prolongation. (a) Representative Cx43 mRNA of rat left ventricle tissues at 120 min after droperidol i.v. with the absence and presence of P6 EA before treatment. (b) Quantitative densitometric analysis of Cx43 mRNA with GADPH as an internal standard. All values are expressed as mean ± SD; ^#^ *P* < 0.05 versus droperidol group (D), *P > 0.05 versus control group (C). C: control group (*n* = 8); D: droperidol group (0.13 mg/kg, *n* = 8); and E: electroacupuncture on P6 + droperidol (*n* = 8).

The results of Cx43 mRNA expression are shown in Figure 2. There was no significant difference between control and droperidol groups (*P* > 0.05), while P6 EA significantly increased Cx43 mRNA (*P* < 0.01 versus droperidol group). Results indicate that Cx43 mRNA expression was increased by P6 EA, and antiemetic dose of droperidol had no significant effects on Cx43 mRNA expression at 120 min.

### 3.3. Effect of P6 EA on the Droperidol-Induced Cx43 Protein Changes

**Figure 3:**
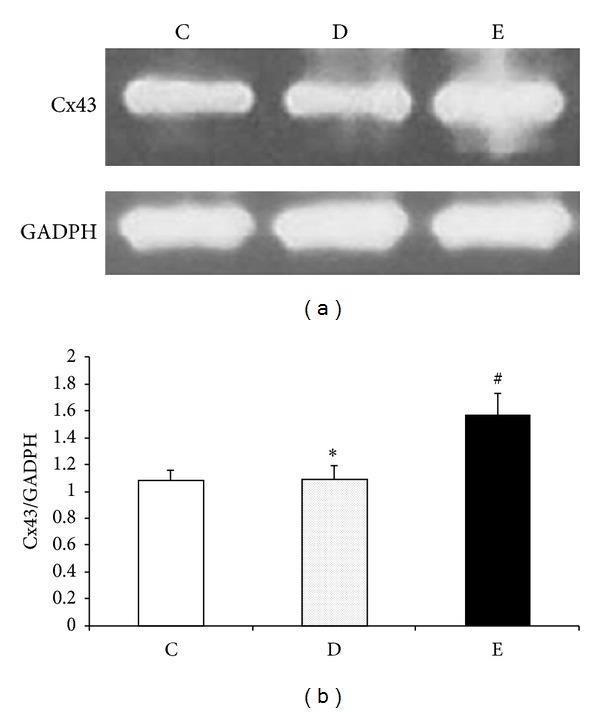
Effects of P6 EA on Cx43 protein expression in a rat model of droperidol-induced QTc prolongation. (a) Cx43 protein expression. Representative Cx43 protein of rat left ventricle tissues at 120 min after droperidol i.v. with the absence and presence of P6 EA before treatment. (b) Quantitative densitometric analysis of Cx43 protein with GADPH as an internal standard. All values are expressed as mean ± SD; ^#^ *P* < 0.05 versus droperidol group (D), *P > 0.05 versus control group (C). C: control group (*n* = 8); D: droperidol group (0.13 mg/kg, *n* = 8); and E: electroacupuncture on P6 + droperidol (*n* = 8).

As shown in Figure 3, Cx43 protein was significantly increased after P6 EA (*P* < 0.05 versus droperidol group), and there was no significant difference between antiemetic dose of droperidol and control groups (*P* > 0.05). Results indicated that Cx43 protein expression was increased by P6 EA, and antiemetic dose of droperidol had no significant effects on Cx43 protein expression at 120 min.

## 4. Discussion

QT prolongation is due to blocking the human ether-a-go-go-related gene (HERG) cardiac potassium channel [5]. The FDA warned droperidol against its serious arrhythmias even at antiemetic “low” (0.625–1.25 mg) antiemetic doses, and more studies are needed to determine the safety and efficacy of droperidol [6]. Based on the formula of human conversion to animal doses recommended by the FDA, the equivalent rat dose of droperidol 1.25 mg was 0.13 mg/kg [7].

With the simulated model of antiemetic prophylaxis in rats, present study indicates that the injection of droperidol (0.13 mg/kg) significantly prolonged QTc interval at 5 min, 10 min, 15 min, and 30 min. Moreover, the peak value was recorded at 10 min. The direct mechanism of the prolonged QTc interval is due to blocking the rapid component of the delayed rectifier potassium current and lengthening cardiac repolarization [8]. However, QTc interval is influenced by multifactors including female gender, old age, heart disease, hypokalemia, and hypomagnesemia [9]. The autonomic nervous system also plays an important role in the modulation of QT interval through the effects on ventricular myocardium repolarization [10], and the QT interval dynamicity could also be used to evaluate the modulation of autonomic neural effects on the ventricular tissues [11]. As shown in Figure 1, the pretreatment with EA at P6 acupoint could prevent the QTc interval prolongation induced by droperidol. The mechanism may be attributed to the ANS modulation activated by P6 EA. Acupuncture at P6 was reported with the increased mean RR interval in ECG through the vagal modulation [12]. From the formula QTc-B = QT/√(RR), EA at P6 might also decrease QTc interval indirectly through increasing RR interval.

P6 is a classic acupoint in traditional Chinese medicine for the treatment of cardiovascular disorders with antiarrhythmic effects. P6 needling was reported to enhance the therapeutic effect for frequent ventricular extrasystole [13]. Laser acupuncture stimulation at the P6 point was reported to increase vagal activity and suppression of cardiac sympathetic nerves [14]. Thus, P6 EA may change the balance of sympathetic and parasympathetic tone and improve the QT prolongation induced by droperidol; however, the molecular mechanism of the cardiac effects remains unclear and may involve ion channels. There were evidences which supported that anesthetics induced gap junctions' reduction, impaired intercellular coupling, and contributed to the occurrence of arrhythmias [15].

Gap junctions are hexagonal protein complexes forming channels between adjacent cells, facilitate intercellular communication, and increase electric conductance, which may contribute to the transmural heterogeneity of repolarization in the normal and the QT interval prolonged heart. Cx43 is the most important subtype and plays an important role in QTc interval. Cx43 was involved in cardiac arrhythmias during ischemia and reperfusion, and EA pretreatment could prevent the arrhythmias through Cx43 pathway. Preservation of Cx43 or preventing dephosphorylation of Cx43 was reported to abbreviate the QT interval [16]. Sun et al. reported that Cx43 preservation could significantly decrease the incidence of ventricular arrhythmias and prolonged the PR interval induced by ischemia [17]. Cx43 also has close relations with acupuncture, and there was high density of Cx43 at acupuncture points [18], and the gap junctional intercellular communication may play an important role in the function of meridians [19]. Cx43 may be an important mediator in the mechanisms of acupuncture and meridians.

The present study demonstrated that antiemetic dose of droperidol had minor effects on Cx43 mRNA and protein expression at 120 min after injection, which suggested the safety of droperidol at antiemetic doses. Pretreatment of P6 EA significantly reduced QTc prolongation induced by droperidol, and this property may be related to the upregulation of Cx43 mRNA and protein, which may contribute to the transmural heterogeneity of repolarization and abbreviate the prolonged QT intervals in droperidol treated hearts.

## 5. Conclusion

The results of rats' models indicate that P6 EA could improve the prolongation of QTc interval induced by droperidol, which may relate to upregulation of Cx43 mRNA and protein. Antiemetic dose of droperidol had minor effects on Cx43 mRNA and protein expression.
